# Effect of Audiovisual Training on Monaural Spatial Hearing in Horizontal Plane

**DOI:** 10.1371/journal.pone.0018344

**Published:** 2011-03-29

**Authors:** Kuzma Strelnikov, Maxime Rosito, Pascal Barone

**Affiliations:** 1 Université Toulouse, CerCo, Université Paul Sabatier, Toulouse France; 2 CNRS, UMR 5549. Faculté de Médecine de Rangueil, Toulouse France; 3 Centre Hospitalier Universitaire, Toulouse, France; McGill University, Canada

## Abstract

The article aims to test the hypothesis that audiovisual integration can improve spatial hearing in monaural conditions when interaural difference cues are not available. We trained one group of subjects with an audiovisual task, where a flash was presented in parallel with the sound and another group in an auditory task, where only sound from different spatial locations was presented. To check whether the observed audiovisual effect was similar to feedback, the third group was trained using the visual feedback paradigm. Training sessions were administered once per day, for 5 days. The performance level in each group was compared for auditory only stimulation on the first and the last day of practice. Improvement after audiovisual training was several times higher than after auditory practice. The group trained with visual feedback demonstrated a different effect of training with the improvement smaller than the group with audiovisual training. We conclude that cross-modal facilitation is highly important to improve spatial hearing in monaural conditions and may be applied to the rehabilitation of patients with unilateral deafness and after unilateral cochlear implantation.

## Introduction

Binaural localization of sounds includes several strategies which are based on interaural difference cues in the sound pressure levels and in the times of arrival of the sound [1]. These abilities are lost in the monaural condition, as demonstrated in patients with unilateral deafness who have problems in spatial localization of sounds [2], [3]. However, some patients with monaural deafness demonstrate very high abilities in sound localization which are close to binaural hearing controls [3]. This suggests that patients have developed a specific strategy probably associated to brain plasticity, which helps to adapt to the monaural condition. Several mechanisms have been proposed to account for monaural sound localization. In monaural conditions, subjects can use spectral cues [3], [4] and the head-shadow effect [5], which corresponds to the attenuation and filtering caused by the head. However, while these cues can account for some performances in monaural sound localization, not all unilateral deaf patients present satisfactory performances [3]. Such impairment is especially accentuated in unilaterally cochlear implanted deaf patients [6], [7]. In this case, patients probably use the information provided by the other sensory modalities (cross-modal compensation), especially the visual channel because visuo-auditory interactions can improve sound localization [8].

Adaptive cross-modal brain plasticity is known to be a common case after sensory loss [9]. We suggest that this plasticity may result from the efficient coupling of the auditory and visual spatial cues in everyday life. Cross-modal facilitation mediated by spatial attention enhances the perceptual salience of stimuli and may be a fundamental operation in multisensory ecological situations [10]. Benefits from multisensory processing can affect a range of different measures from reaction times, detection rates or accuracy of stimulus identification as well as learning effects on stimulus processing [11]. If so, special techniques of audiovisual training [12] can be elaborated to improve spatial localization of sounds with one ear.

Recent psychophysical studies have shown that audiovisual training can increase the rate of learning and can improve perceptual performance in the auditory or visual modality alone [13], [14], [15]. Visual information may provide a strong positive feedback that facilitates the “decoding” of auditory cues because the primary auditory cortex can retain long-term memory traces about the behavioural significance of sounds [16]. A possible neural underpinning of this neural feedback may lie in the direct heteromodal connections between sensory areas of different modalities [17], [18].

**Figure 1:**
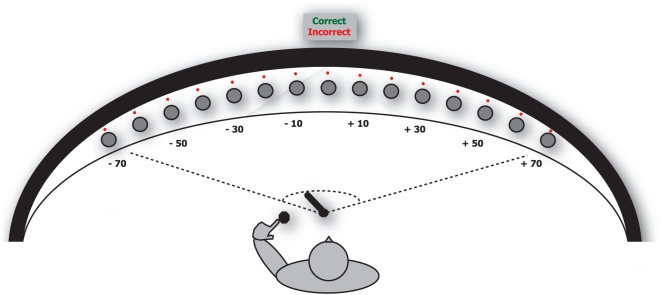
Schematic illustration of the experimental setup. Fifteen loudspeakers presented sound stimuli from different azimuthal directions. Loudspeakers are located in front of the subject on a semicircle device of a radius of 0.5 m. With a special knob, the subject turned a laser beam originating from the centre of a semicircle of loudspeakers and confirmed the position of the laser beam pressing the button. In response to the pressed button, the laser beam position was registered by laser detectors in the semicircle of loudspeakers.

The aim of our study was to find out whether enhanced audiovisual integration induced by training can improve the localization of a sound source in monaural conditions. To test our hypothesis, we trained one group of subjects in an auditory-only protocol, another group of subjects with spatially and temporally congruent audiovisual stimuli (Figure 1). Besides, to check whether the observed audiovisual effect was different from behavioural feedback, we trained the third group of subjects with a visual feedback paradigm. For each group of subjects, we compared their spatial hearing in the auditory only modality before and after five daily training sessions.

## Results

### Pre-training performances

**Figure 2:**
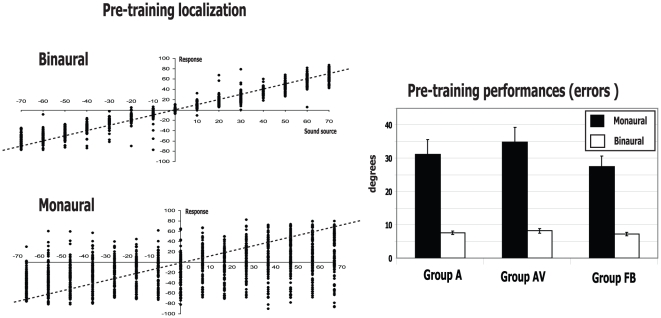
Performances of the subjects in the binaural and monaural conditions. On the left, the horizontal axis represents the azimuthal positions of loudspeakers, the vertical axis represents the azimuthal response of the subjects. On the right, the mean pre-training responses collapsed across the positions are shown for each group of subjects. The dashed line is the ideal performance curve in this case (e.g., the sound source at 60° corresponds to the response at 60°).

Three days before the training session, all the subjects were tested in bi- and monaural conditions for auditory localisation of sounds. When combining all the subjects, the binaural performances were relatively precise (see Figure 2) with mean unsigned errors of 7.7±0.3°. Such performance level is similar to that reported in previous studies using, for example, a head orientation response (see Middlebrooks and Green 1991 for a review) but which is less than reported with a similar laser beam pointer apparatus [19]. When the subjects' performance is compared across the groups (A, AV and FB), there were no statistical differences between the three groups in the pre-training sound localization abilities (bootstrap analysis). In monaural condition, all subjects showed a dramatic alteration ins their abilities to localize a sound source in azimuth. As previously reported [3], plugging one ear induced a shift of responses toward the unplugged side (see Figure 2). In terms of accuracy of localization, we observed a strong increase in the mean unsigned error of the subjects compared to binaural conditions (all subjects combined, mean error 30.4±1.4°, p<0.05, Figure 2 right). A bootstrap statistical analysis revealed the same amount of deficit when comparing the three groups (groups A, AV and FB, Figure 2) in this pre-training session.

### Post-training performances

In all groups, the subjects went through a daily session of monaural sound localization during 5 consecutive days. A direct comparison between pre- and post-training performance when the sound was presented alone, showed that the accuracy of the subjects increased after 5 days of practice. However, in spite of a daily training, the subjects never reach the performance level observed in binaural conditions during the pre-training test and in all groups the unsigned errors (in degree) remained statistically higher than that observed in the normal situation (bootstrap).

**Figure 3:**
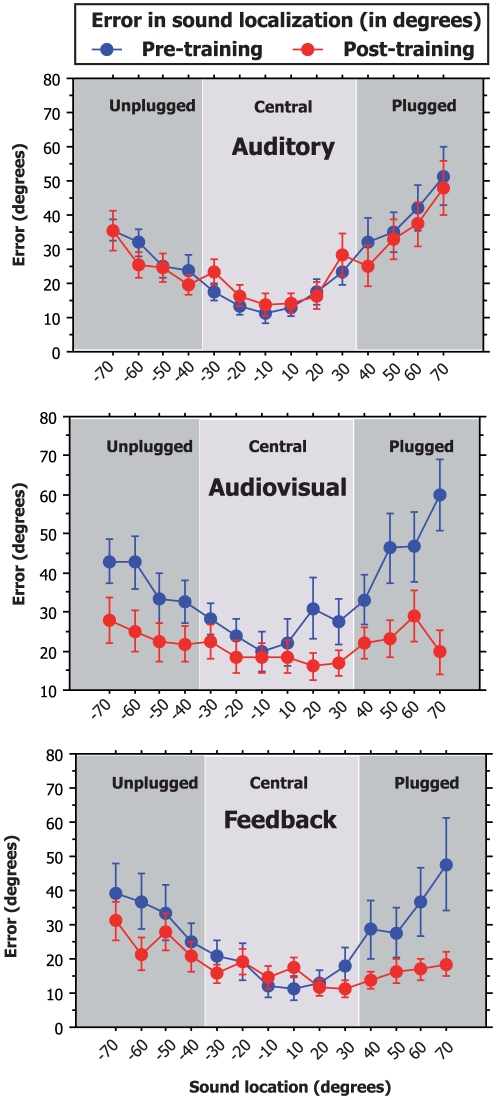
Improvement after training in total and per side. Improvement is presented as the difference in error (deviation from the sound source) before and after training. Error bars represent bootstrap bias-corrected and accelerated confidence intervals (p<0.05).

The improvement in sound localization varies according to the conditions of practice (auditory only, audiovisual, with feedback) and according to the side of stimulation with respect to the plugged ear. When comparing the unsigned error before and after training (Figure 3), one can see that this improvement is the smallest for the auditory training group, much higher for the group with feedback, and even higher for the audiovisual training group. For example, in the A-only group, the subjects present a global reduction of about 0.9° (±0.1°) in their errors localizing a sound source in azimuth. Such decrease in unsigned errors is small but significant (bootstrap) when comparing the pre- and post-training values.

In the group which received a visual indication of their performance accuracy (group FB), subjects also present an overall improvement in their sound localization performances expressed as a 10.8° (±0.1°) reduction in their mean unsigned error of sound localization.

**Figure 4:**
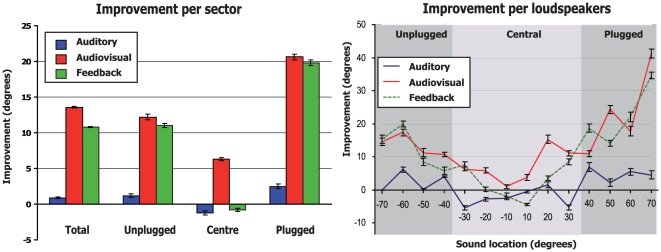
Improvement after training per side per loudspeaker. Improvement is presented as the difference in error (deviation from the sound source) before and after training. Error bars represent bootstrap bias-corrected and accelerated confidence intervals (p<0.05).

In the audiovisual group (group AV), a spatially congruent visual stimulation was presented simultaneously to the sound. After the 5 training sessions, when tested in auditory alone conditions, this group showed the highest improvement in monaural sound localization with a global decrease in unsigned errors of 13.6° (±0.1°). Such increase in accuracy is statistically greater than that observed in the FB and A-only groups (respectively 10.8 and 0.9°, p<0.05 bootstrap). Of importance is that such an improvement by audiovisual training can be observed for all the spatial fields in the azimuth. The highest reduction in spatial errors was observed when the sound was presented in the 30–70° ispilateral to the plugged ear (20.6°±0.4° reduction). The improvement was the smallest in the −30°/+30° central region (6.3°±0.2° decrease) and intermediate when the sound appeared in the side ipsilateral to the unplugged ear (12.2°±0.4°). In the A-only and the FB groups, the amelioration of the performances of the subjects is also the strongest for sound location in the azimuth ipsilateral to the plugged ear. In the A-only and FB group the subjects tend to be worse in localizing the auditory stimuli when it appears in the central 30 degrees from the central fixation point on each side. On the opposite, in the AV group, the subjects showed a fairly significant improvement in sound localization for sound located in the central region. This can be also seen in Figure 4 where the pre-post-training difference in error is presented per loudspeaker. To clarify these differences in localizing sounds at the central positions, we analyzed the performance of the subjects with respect to the correct left/right discrimination. In the AV group, we observed a significant reduction (13±7%, p<0.05) in the lateralization errors when comparing pre- and post-training performance in auditory conditions for the central position (10–30° on both sides). On the opposite, both A-only and FB groups did not improve their performance in localizing the correct side of the sound (p>0.05). Such results can explain, at least partly, the absence of amelioration of sound localization when expressed in degrees.

Finally, we compared the performance of the audiovisually trained group for the audiovisual trials in the beginning of the training and in the end. This analysis was performed to check if the subjects were guided by the visual cues to localize the sound source. The errors during the audiovisual stimulation were minimal (4.5±0.3°, p<0.05) and were much lower than that observed for the same group in the binaural pre-training session (p>0.5). Further, no significant difference was found for any loudspeaker in the comparison of the training days (p>0.7). Such analysis suggests that the subjects were highly influenced by the visual stimuli to localize the sound but failed to reveal an improvement due to the training in the visual modality.

To conclude, we observed the highest significant improvement in monaural sound localization when the sound is simultaneously accompanied by the visual cue; such improvement can be obtained for all azimuth location of the sound source.

### Changes in perceptual sensitivity

**Figure 5:**
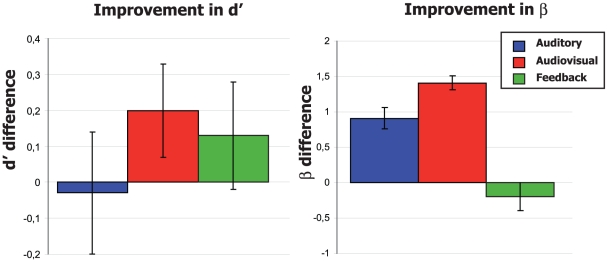
Changes of d′ and ß due to training. Differential values after and before training are presented. Error bars represent bootstrap bias-corrected and accelerated confidence intervals (p<0.05).

We used signal detection measures to separate perceptual (d′) and decision-level (ß) effects. On the basis of the similar performances between groups during the pre-training test, we searched for an effect of the training protocols (A, AV, or FB) on the evolution of the d′ values with the hypothesis of a larger increase of the perceptual sensitivity following the bimodal training. As explained in the method section, we considered as “hits” the responses located within 5° from the centre of the sound source. This method for the hits corresponds to the one applied in the feedback group to indicate the correct response during the experiment, thus we can compare directly the d′ values of all our groups. The direct comparison of pre- and post-training values of d′ (Figure 5) did not reveal a significant improvement for the A-only and feedback groups. On the opposite, when considering the audiovisual training group we observed a statistically increase in d′ values (bootstrap analysis) which can be interpreted as a facilitatory perceptual effect of the bimodal training on monaural sound localization.

It is worth mentioning that the different training protocols are also expressed by different variations in the decision criteria of the subjects when pre- and post-training data are compared (Figure 5). Subjects in both the A-only and AV groups present a significant increase in the decision criteria, while we did not observe a variation in the Feedback group. Altogether, this suggests that the training procedures induced different strategies to localize accurately the sound by the subjects.

## Discussion

### Monaural sound localization and practice

In normal binaural conditions, sound localization in the horizontal plane is performed by computing the differences in intensity level or time of arrival of the sound (ILD and ITD respectively) that are present between the two ears [1]. In case of monaural conditions, sound localization can be performed only by using the spectral cues provided by pinna filtering which amplifies or attenuates differently the frequencies according to the azimuthal sound sources [3]. In this case, the performances are poor in term of precision and the perception of sounds presented from the plugged ear is displaced toward the unplugged functional ear. Using an active laser pointing, we have replicated such results and we observed a large error in horizontal sound localization, over 30° of error, which correspond to that previously reported [20]. After a daily practice of sound localization with an ear plugged, we observed some reduction in the spatial errors of the subjects which is highly dependent on the protocol showing a significant advantage for a bimodal visuo-auditory training.

There are numerous evidences that normal hearing subjects can learn to localize a sound source when the spatial cues are experimentally modified [21], [22], either following plugging one ear [3], [21], [23] or after altering the spectral cues [24], [25]. In our experimental design, a moderate daily training of monaural sound localization is not sufficient to restore the same level of performance observed during natural binaural stimulation. We report only a weak improvement as a reduction of a few degrees in spatial error. Other studies using a chronic ear plug during one or several days have reported a higher rate of recovery of sound localization [3], [21]. Probably, during a continuous earplug, subjects are able to interact with the environment and therefore can use the visual information to recalibrate the altered spatial cues with the sound source location. The role of visual inputs in spatial auditory adaptation has been clearly demonstrated using modified vision [26] and is also present in our study as the simultaneous presentation of a spatially congruent visual stimulus leads to the greater amount of improvement.

### Facilitation of auditory adaptation

Several hypotheses have been proposed to explain how subjects can adapt to the altered interaural cues induced by the earplug including internal representation [23]. During the unisensory A-only training, the only criterion available for the subjects to differentiate between the sounds coming from the unplugged and plugged sides was the possible effect of head shadow and/or pinna cues [5], [27]. The Head Shadow effect leads to the difference both in the intensity and spectral characteristics of the perceived sound because different frequencies are absorbed differently by the head [28], [29]. Such effect can be efficiently used only when the sounds to localized are of the same intensity such as in the present experiment. Further, a strategy based on such effect could also explain the higher improvement in all groups for auditory stimuli presented in peripheral location compared to central presentations.

However, in the AV and FB groups, additional mechanisms have to be considered because these subjects present a much higher level of adaptation to the altered binaural cues when compared to the A-only group. Perceptual learning [30] and associated brain plasticity mechanisms are probably participating to the amelioration of sound localization of these subjects. Perceptual learning corresponds to the improvement in perceptive performance induced by repeated sensory practice. The implication of multimodal perceptual learning in our protocol is reinforced by the observation of an increase in d′ values that reflect strict perceptual enhancement induced by the training. Such an increase in d′ values excludes the possibility that the visual stimulus is affecting the performance at a cognitive level while it does induce decisional changes as shown independently by the higher ß values. The role of feedback and top-down mechanisms have been shown to have a strong impact on perceptual learning [31]. In the FB group, in half of trials the subjects received a visual indication of the accuracy of their response. While the feedback signal in case of incorrect responses (a spatial error greater than 5°) does not provide to the subjects a magnitude of their mislocalization, subjects were able to use this signal to recalibrate the altered spatial cues.

One conceptual question which emerges from our results concerns the theoretical differences between the “feedback” inputs and multisensory interactions in the present conditions. In the group that received a bimodal visuo-auditory stimulation in half of the trials (AV group), the improvement can be also interpreted as resulting from the feedback mechanisms provided by the azimuth and time congruent LED. The temporal order of feedback inputs is important in the efficiency of perceptual learning [32]; the temporal and spatial congruencies serve as key features to obtain the maximal gain from multisensory interaction [11]. However, there are some important peculiarities that suggest that multisensory interaction might constitute different mechanisms supported by different neuronal processes and/or structures from the feedback part of our study. Firstly, the multisensory paradigm has induced an improvement in perceptual sensitivity (d′) and a modification of the decision criteria (ß). On the opposite, the feedback training did not influence neither the perceptual sensitivity nor the decisional criteria of the subjects. Further, the feedback training did not improve sound lateralization for the more misleading positions (10–30° from the centre), while the audiovisual training did reduce significantly the errors in allocating the sound to the correct hemi-field. Such result might account for the greater absolute errors observed in the feedback group when considering these positions. It should be noted that when the changes in both d′ and ß are present, their attribution is rather difficult as they could be related to increased sensitivity, cognitive bias, or both. Given that sound localization and auditory left/right lateralization involve probably different auditory structures (see [33], [34], [35]) we can suspect that the improvement obtained in our study by the AV and FB groups results from different mechanisms. Moreover, additional studies using variable temporal and spatial mismatch in the visuo-auditory stimuli need to be performed to dissociate clearly the benefits due to Feedback mechanisms from those multisensory integration.

### Role of multisensory training

There is a large body of evidence for the importance of synergy between sensory modalities in our global perception and the associated behaviour [11]. Indeed, simultaneous polysensory stimulation results in qualitative percept distinct from those derived from a single uni-sensory stimulus [36]. Under specific context of congruency, multisensory integration results in perceptual improvements in various tasks, from simple detections to complex discriminations and memory [37], [38]. In addition, the role of multisensory interactions had been extended to visual learning and adaptation (reviewed in [39], [40]). In such cases, when comparing uni- and multisensory training, it has been shown that a multimodal practice induced a significantly better learning both in term of performance and of speed rate [14]. In addition, multisensory learning improves various types of sensory processing, such as visual motion detection [41] or visual temporal order judgment [42] and even auditory speech comprehension [43]. Our results in the present study show that benefits of crossmodal perceptual learning can be extended to auditory perception such as to sound localization. By presenting a temporally and spatially (in azimuth) congruent visual cue, subjects present a significant larger improvement in monaural localization, a result in line with the rule that multisensory perceptual learning depends on the congruency of the two sensory stimuli [41], [44].

### Neuronal mechanisms of multisensory training

Our results showed that the repetitive presentation of the visual stimulus in the temporal and spatial congruence with the auditory stimulus can improve the performance for the auditory stimulus presented in isolation. Such results imply a convergence of the visual and auditory spatial representations in the brain. Several studies have pointed out the role of the tectum in merging auditory and visual spatial maps (see [45] for a review). Further, these modality specific maps are highly interdependent and any alterations of the visual or auditory modality during the development have a great impact on the spatial representation of the spared modality in the superior colliculus [46], [47], [48]; the mechanisms of sound localization are supported by a large network of subcortical and cortical regions [49], [50], [51]. Among these set of cortical areas, several studies have shown the role of the primary auditory cortex A1 in spatial hearing [33], [35], [52], [53]. Of interest for the present study, it has been shown that A1 contains strictly monaural cells that derived azimuth sensitivity for sound source from spectral cues [54], which are probably important for monaural sound localization [55]. Thus, a crucial question concerns the implication of the early stages of auditory processing in the improvement of monaural sound localization during visuo-auditory training. Recent studies in the ferret suggest an implication of A1 because after alteration of binaural cues, ferrets can recalibrate a sound source location by using visual cues [56], a mechanism that involves the primary auditory cortex [53].

In case of uni-sensory protocols, it has been proposed that perceptual learning is expressed by plastic changes that can occur at early cortical stages of sensory processing [57]. In the visual domain, in both animals [58], [59], [60] and human [61], perceptual learning induces modification of neuronal properties at the level of V1, the primary visual cortex. Similarly, both anatomical [17], [62], [63], [64] and electrophysiological animal studies [65], [66] have shown that the early stages of sensory processing, including V1 [67], are involved in multisensory processing [68]. In humans the implication of early unimodal sensory areas has been similarly shown during multisensory processing [69], [70], [71], [72]. Furthermore, in chronically blindfold subjects, intense Braille reading training induces crossmodal modifications at the level of the primary visual cortex [73] suggesting that crossmodal perceptual learning and multisensory interactions could share some common cortical network [74]. All together, it suggests that the improvement of monaural sound localization performance during the visuo-auditory training could be supported by the direct heteromodal connections that directly link visual and auditory areas [17]. The auditory cortex, in particular the caudal auditory areas involved in spatial processing [75], [76], is receiving direct inputs from the pre-striate cortex [63] originating specifically from the representation of the peripheral visual field. Such specificity in this visuo-auditory connection could account for our observation of a higher post-training improvement in localizing sound sources located over 30° of eccentricity. Thus, one could suggest that the visual presentation concomitant to the sound will reinforce the role of monaural spectral processing in A1 through Hebbian mechanisms [77] via the direct visual projections to the auditory cortex. However, we cannot exclude that the influence of multisensory training on monaural sound localization can be mediated in addition by a top-down influence originating from multisensory high-order areas. The caudal auditory cortex is receiving non-auditory inputs including visual, from the temporal, parietal and frontal lobes [78], [79], [80], which can participate in the recalibration of the sound source throughout the training sessions.

### Implication for rehabilitation of patients with sensory loss

There is some data, though not related to spatial hearing, that ecological visual cues play a very important role in patients with unilateral cochlear implants (CI) helping them to restore the auditory modality. We have shown that in post-lingual CI recipient, patients maintain the high skill in lip-reading acquired during the prolonged period of deafness, even after several years of auditory recovery [81], [82]. Our previous observations suggest a synergetic perceptual facilitation involving the visual and the recovering auditory modalities, which can be observed both at the behavioural [81] and brain levels [83] in the speech domain. Furthermore, multisensory perceptual learning is improving speech comprehension in normal hearing subject tested with a degraded auditory information using a simulation of a cochlear implant [43]. Based on the present results, we can propose that the sound localization deficit observed in unilaterally CI deaf patients [6], [84] (Grantham et al 2004) could be reduced by intense visuo-auditory training. Such strategy of multisensory stimulation has been shown to be efficient in patients suffering of visual hemineglect and hemianopsia [85], [86].

## Materials and Methods

### Subjects

Eighteen normally-hearing subjects (mean age 25, range 20–40) participated in the protocol. They were divided into 3 groups with no distinction of age and gender between the groups (3 men and 3 women per group). All subjects reported no auditory or neurological disease and had normal or corrected to normal vision. All participants gave their full-informed consent prior to their participation in this study in accordance with the Declaration of Helsinki (1968). The study was approved by the local research ethics committee (Comite Consultatif de Protection des Personnes dans la Recherche Biomédicale Toulouse II Avis N°2-03-34/Avis N°2). The subjects were financially compensated for their participation.

### Experimental set-up

The experiment was conducted in a dark soundproof anechoic room. A subject sat on a chair with his chin stabilized on a special framework (UHCOTech HeadSpot). During the experiment, the subjects were asked to fixate upon a green light-emitting diode in front of them which corresponded to the central loudspeaker. The study was realized in monaural conditions; one ear of the subject was plugged with an ear plug (average noise reduction 30 dB) and covered with an ear muff (average noise reduction 20 dB). The opposite muff was taken off, a sponge was glued to the resulting free end of the muffing device and it was comfortably placed behind the subject's open ear during the experiment.

The apparatus consisted of 15 piezoelectric loudspeakers arranged horizontally in semicircle with a radius of 0.5 m in front of the subject, the subject being in the centre of the semicircle (Figure 1). Loudspeakers in the semicircle were masked by a black acoustically transparent fabric so that the subject could not visually distinguish them. They were mounted on a plastic support that was held in place by 4 wooden stands fixed to the table. The angular positions of the loudspeakers were 70°, 60°, 50°, 40°, 30°, 20°, 10° with respect to the central loudspeaker (0°). Right above the centre of each loudspeaker, a red light-emitting diode (LED) was fixed.

Having perceived the sound from a loudspeaker (accompanied sometimes by the corresponding LED, see below), the subject had to indicate the source of the stimulation with a laser beam. This beam was projected from a rotating emitter in the centre of the semicircle of loudspeakers which can be manipulated by the subject through a manual knob. A home-made device, using a numerical potentiometer was allowed to record the position of the laser beam on the semicircle with a precision of 0.3°. A knob for rotating the laser emitter was on both the right and the left sides of the support so that the subjects could use either hand. Near each knob, there was a button to confirm the response. The subjects turned the knob with their preferred hand and pressed the button on the other side with the other hand. At time the response button was pressed, the laser position in degrees was registered. Having confirmed the response with the button, the subject repositioned the laser beam at the centre of the semicircle and waited for the next trail to start. The inter-trials time interval was random in the interval of 0.5–1.5 sec.

Auditory stimuli were the rectangular white noise (0,1–22 kHz) generated by Adobe Audition 3.0 lasting 50 ms and presented at the intensity of 60 dB SPL (measured at the centre of the semicircle of loudspeakers). Visual stimuli (red LEDs) of same duration (50 ms) were delivered simultaneously to the sound in cases of visuo-auditory conditions (see below). The LEDs were located above each loudspeaker (and above the black fabric covering the loudspeakers).

### Experimental protocols

Three groups of 6 subjects participated to this protocol during which they underwent a testing session once a day during five consecutive days. Three days before (pre-training session), subjects had to perform the task in binaural and in monaural conditions to get familiar with the apparatus and to asses their *pre-training* sound localization abilities. The subjects were firstly engaged in 15 trials to familiarize them with the device, then 5 trials per loudspeaker were presented binaurally (75 random trials). Then the presentation of the monaural (to the left ear) auditory stimulation followed with 10 trials per loudspeaker resulting in 150 random trials. Having perceived the sound from a loudspeaker, the subject had to indicate the source of the stimulation with a laser beam as explained above. The pre-training session as well as the training sessions lasted about 1 hour including 2 pauses of 5 minutes each.

During the training sessions, the subjects were divided into three comparable groups. In one group, the conditions of stimulation were auditory only (Group A). In the second group, auditory stimuli were accompanied in half of the cases by an azimuth spatially congruent visual LED (Group AV). Audiovisual trials in this second group were presented randomly among auditory stimuli. In a third group of 6 subjects, stimulation was only auditory but in half of the cases a feedback was given to the subjects on the accuracy of their performance (Group FB). After pressing the button a small screen fixed above the central loudspeaker indicated “correct” or “incorrect”. A “Correct” message was given when the response was ±5° from the centre of the correct loudspeaker. If the response was outside this 5° range, the “incorrect” indication was presented. All the experimental conditions were similar to the ones of the auditory group (Group A).

Each day during 5 consecutive days, subjects were presented a session of 20 trials per loudspeaker (300 random trials). The performance of the subjects of all groups were analyzed and compared before (pre-training) and after this 5 days practice (post-training) during an auditory-alone presentation. Thus, by comparing the pre- and post-training performance, in Group A we assessed the effect of auditory practice, while in group AV we could observe the effect of audiovisual training on spatial monaural hearing in the horizontal plane.

### Data analysis

Direct comparisons of the post- and pre-training performance, as well as between groups, were performed using the bootstrap method with bias-corrected and accelerated confidence intervals [87]. The effect was considered to be significant if there was no overlapping of confidence intervals at p<0.05.

First, we considered the difference in unsigned error (deviance of the response from the source of the sound in degrees) before and after training for each group. For each loudspeaker, the errors were re-sampled 60 times, then we calculated the mean post- pre-training difference for each sample and re-sampled the difference 10000 times to obtain confidence intervals per loudspeaker.

We also calculated the post- pre-training difference after dividing the semicircle into three sectors: the “plugged sector” corresponding to the responses to sound locations at 70°, 60°, 50°, 40°, and 30° on the side ipsilateral to the plugged ear, the central sector corresponding to the positions at 10° and 20° on both sides, the “unplugged” sector that encompass the locations at 70°, 60°, 50°, 40°, and 30° ipsilateral to the unplugged ear. For each sector, we re-sampled the error 300 times, calculated the mean post- pre-training difference for each sample and re-sampled the difference 10000 times to obtain confidence intervals per sector.

Finally, we applied SDT to analyse the performance of the subjects [88] to separate decisional bias from perceptual mechanisms. In this case, we considered as hits the responses located ±5° from the centre of the loudspeaker. We have chosen this value because it corresponded to the values applied in the feedback group (Group FB) to indicate a correct response of the subject. Then we calculated the post- pre-training differences in d′ and ß per sector. The differences between d′ and ß were calculated for each subject per loudspeaker and then re-sampled 10000 times to obtain confidence intervals per sector. The d′ and ß values were calculated according to the Matlab formulas:
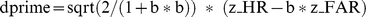


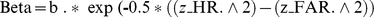
where HR is Hit Rate, FAR - False Alarm Rate and b is the input. Here, z_HR = −sqrt(2) * erfcinv(2*HR), where erfcinv is the inverse complementary error function. False alarm rate was determined as the response to the given loudspeaker when the sound was emitted elsewhere.

## References

[1] Middlebrooks JC, Green DM (1991). Sound localization by human listeners.. Annu Rev Psychol.

[2] Colletti V, Fiorino FG, Carner M, Rizzi R (1988). Investigation of the long-term effects of unilateral hearing loss in adults.. Br J Audiol.

[3] Slattery WH, Middlebrooks JC (1994). Monaural sound localization: acute versus chronic unilateral impairment.. Hear Res.

[4] Shub DE, Carr SP, Kong Y, Colburn HS (2008). Discrimination and identification of azimuth using spectral shape.. J Acoust Soc Am.

[5] Van Wanrooij MM, Van Opstal AJ (2004). Contribution of head shadow and pinna cues to chronic monaural sound localization.. J Neurosci.

[6] Luntz M, Brodsky A, Watad W, Weiss H, Tamir A (2005). Sound localization in patients with unilateral cochlear implants.. Cochlear Implants Int.

[7] Nava E, Bottari D, Bonfioli F, Beltrame MA, Pavani F (2009). Spatial hearing with a single cochlear implant in late-implanted adults.. Hear Res.

[8] Bolognini N, Frassinetti F, Serino A, Ladavas E (2005). “Acoustical vision” of below threshold stimuli: interaction among spatially converging audiovisual inputs.. Exp Brain Res.

[9] Bavelier D, Neville HJ (2002). Cross-modal plasticity: where and how?. Nat Rev Neurosci.

[10] McDonald JJ, Teder-Salejarvi WA, Hillyard SA (2000). Involuntary orienting to sound improves visual perception.. Nature.

[11] Stein BE, Meredith MA (1993). The Merging of the Senses.

[12] Ladavas E (2008). Multisensory-based approach to the recovery of unisensory deficit.. Ann N Y Acad Sci.

[13] Frassinetti F, Bolognini N, Bottari D, Bonora A, Ladavas E (2005). Audiovisual integration in patients with visual deficit.. J Cogn Neurosci.

[14] Seitz AR, Kim R, Shams L (2006). Sound facilitates visual learning.. Current Biology.

[15] Lippert M, Logothetis NK, Kayser C (2007). Improvement of visual contrast detection by a simultaneous sound.. Brain Res.

[16] Weinberger NM (2004). Specific long-term memory traces in primary auditory cortex.. Nat Rev Neurosci.

[17] Cappe C, Barone P (2005). Heteromodal connections supporting multisensory integration at low levels of cortical processing in the monkey.. Eur J Neurosci.

[18] Cappe C, Morel A, Barone P, Rouiller EM (2009). The thalamocortical projection systems in primate: an anatomical support for multisensory and sensorimotor interplay.. Cereb Cortex.

[19] Lewald J, Ehrenstein WH (1998). Auditory-visual spatial integration: a new psychophysical approach using laser pointing to acoustic targets.. J Acoust Soc Am.

[20] Butler RA (1987). An analysis of the monaural displacement of sound in space.. Percept Psychophys.

[21] Kumpik DP, Kacelnik O, King AJ (2010). Adaptive reweighting of auditory localization cues in response to chronic unilateral earplugging in humans.. J Neurosci.

[22] Wright BA, Zhang Y (2006). A review of learning with normal and altered sound-localization cues in human adults.. Int J Audiol.

[23] Musicant AD, Butler RA (1984). The psychophysical basis of monaural localization.. Hear Res.

[24] Hofman PM, Van Opstal AJ (1998). Spectro-temporal factors in two-dimensional human sound localization.. J Acoust Soc Am.

[25] Van Wanrooij MM, Van Opstal AJ (2005). Relearning sound localization with a new ear.. J Neurosci.

[26] Zwiers MP, Van Opstal AJ, Paige GD (2003). Plasticity in human sound localization induced by compressed spatial vision.. Nat Neurosci.

[27] Van Wanrooij MM, Van Opstal AJ (2007). Sound localization under perturbed binaural hearing.. J Neurophysiol.

[28] Ison JR, Agrawal P (1998). The effect of spatial separation of signal and noise on masking in the free field as a function of signal frequency and age in the mouse.. J Acoust Soc Am.

[29] Darwin CJ, Hukin RW (2004). Limits to the role of a common fundamental frequency in the fusion of two sounds with different spatial cues.. J Acoust Soc Am.

[30] Goldstone RL (1998). Perceptual learning.. Annu Rev Psychol.

[31] Seitz A, Watanabe T (2005). A unified model for perceptual learning.. Trends Cogn Sci.

[32] Hervais-Adelman A, Davis MH, Johnsrude IS, Carlyon RP (2008). Perceptual learning of noise vocoded words: effects of feedback and lexicality.. J Exp Psychol Hum Percept Perform.

[33] Jenkins WM, Masterton RB (1982). Sound localization: effects of unilateral lesions in central auditory system.. J Neurophysiol.

[34] Kavanagh GL, Kelly JB (1987). Contribution of auditory cortex to sound localization by the ferret (Mustela putorius).. J Neurophysiol.

[35] Heffner HE, Heffner RS (1990). Effect of bilateral auditory cortex lesions on sound localization in Japanese macaques.. J Neurophysiol.

[36] Welch RB, Warren DH, Boff KR, Kaufman L, Thomas JP (1986). Intersensory interactions.. Handbook of perception and human performance.

[37] Lehmann S, Murray MM (2005). The role of multisensory memories in unisensory object discrimination.. Brain Res Cogn Brain Res.

[38] Lovelace CT, Stein BE, Wallace MT (2003). An irrelevant light enhances auditory detection in humans: a psychophysical analysis of multisensory integration in stimulus detection.. Brain Res Cogn Brain Res.

[39] Shams L, Seitz AR (2008). Benefits of multisensory learning.. Trends Cogn Sci.

[40] Shams L, Kim R (2010). Crossmodal influences on visual perception.. Phys Life Rev.

[41] Kim RS, Seitz AR, Shams L (2008). Benefits of stimulus congruency for multisensory facilitation of visual learning.. PLoS One.

[42] Alais D, Cass J (2010). Multisensory perceptual learning of temporal order: audiovisual learning transfers to vision but not audition.. PLoS One.

[43] Kawase T, Sakamoto S, Hori Y, Maki A, Suzuki Y (2009). Bimodal audio-visual training enhances auditory adaptation process.. Neuroreport.

[44] Beer AL, Watanabe T (2009). Specificity of auditory-guided visual perceptual learning suggests crossmodal plasticity in early visual cortex.. Exp Brain Res.

[45] Knudsen EI, Brainard MS (1995). Creating a unified representation of visual and auditory space in the brain.. Annu Rev Neurosci.

[46] King AJ, Schnupp JW, Carlile S, Smith AL, Thompson ID (1996). The development of topographically-aligned maps of visual and auditory space in the superior colliculus.. Prog Brain Res.

[47] Withington-Wray DJ, Binns KE, Keating MJ (1990). The developmental emergence of a map of auditory space in the superior colliculus of the guinea pig.. Brain Res Dev Brain Res.

[48] Stein BE, Perrault TJ, Stanford TR, Rowland BA (2009). Postnatal experiences influence how the brain integrates information from different senses.. Front Integr Neurosci.

[49] Middlebrooks JC, Xu L, Furukawa S, Macpherson EA (2002). Cortical neurons that localize sounds.. Neuroscientist.

[50] Clarey JC, Barone P, Imig TJ, Fay R, Popper A (1992). Physiology of Thalamus and Cortex.. The Mammalian Auditory Pathway: Neurophysiology.

[51] King AJ, Schnupp JW, Doubell TP (2001). The shape of ears to come: dynamic coding of auditory space.. Trends Cogn Sci.

[52] Malhotra S, Hall AJ, Lomber SG (2004). Cortical control of sound localization in the cat: unilateral cooling deactivation of 19 cerebral areas.. J Neurophysiol.

[53] Nodal FR, Kacelnik O, Bajo VM, Bizley JK, Moore DR (2010). Lesions of the auditory cortex impair azimuthal sound localization and its recalibration in ferrets.. J Neurophysiol.

[54] Samson FK, Clarey JC, Barone P, Imig TJ (1993). Effects of ear plugging on single-unit azimuth sensitivity in cat primary auditory cortex. I. Evidence for monaural directional cues.. J Neurophysiol.

[55] Neff WD, Casseday JH (1977). Effects of unilateral ablation of auditory cortex on monaural cat's ability to localize sound.. J Neurophysiol.

[56] King AJ (2009). Visual influences on auditory spatial learning.. Philos Trans R Soc Lond B Biol Sci.

[57] Gilbert CD, Sigman M, Crist RE (2001). The neural basis of perceptual learning.. Neuron.

[58] Schoups A, Vogels R, Qian N, Orban G (2001). Practising orientation identification improves orientation coding in V1 neurons.. Nature.

[59] Li W, Piech V, Gilbert CD (2004). Perceptual learning and top-down influences in primary visual cortex.. Nat Neurosci.

[60] Hua T, Bao P, Huang CB, Wang Z, Xu J (2010). Perceptual learning improves contrast sensitivity of V1 neurons in cats.. Curr Biol.

[61] Schwartz S, Maquet P, Frith C (2002). Neural correlates of perceptual learning: a functional MRI study of visual texture discrimination.. Proc Natl Acad Sci U S A.

[62] Falchier A, Clavagnier S, Barone P, Kennedy H (2002). Anatomical evidence of multimodal integration in primate striate cortex.. J Neurosci.

[63] Falchier A, Schroeder CE, Hackett TA, Lakatos P, Nascimento-Silva S (2010). Projection from visual areas V2 and prostriata to caudal auditory cortex in the monkey.. Cereb Cortex.

[64] Rockland KS, Ojima H (2003). Multisensory convergence in calcarine visual areas in macaque monkey.. Int J Psychophysiol.

[65] Fu S, Fan S, Chen L (2003). Event-related potentials reveal involuntary processing of orientation changes in the visual modality.. Psychophysiology.

[66] Kayser J, Tenke CE, Gates NA, Bruder GE (2007). Reference-independent ERP old/new effects of auditory and visual word recognition memory: Joint extraction of stimulus- and response-locked neuronal generator patterns.. Psychophysiology.

[67] Wang Y, Celebrini S, Trotter Y, Barone P (2008). Visuo-auditory interactions in the primary visual cortex of the behaving monkey: electrophysiological evidence.. BMC Neurosci.

[68] Schroeder CE, Foxe J (2005). Multisensory contributions to low-level, ‘unisensory’ processing.. Curr Opin Neurobiol.

[69] Giard MH, Peronnet F (1999). Auditory-visual integration during multimodal object recognition in humans: a behavioral and electrophysiological study.. J Cogn Neurosci.

[70] Foxe JJ, Morocz IA, Murray MM, Higgins BA, Javitt DC (2000). Multisensory auditory-somatosensory interactions in early cortical processing revealed by high-density electrical mapping.. Brain Res Cogn Brain Res.

[71] Molholm S, Ritter W, Murray MM, Javitt DC, Schroeder CE (2002). Multisensory auditory-visual interactions during early sensory processing in humans: a high-density electrical mapping study.. Brain Res Cogn Brain Res.

[72] Sperdin HF, Cappe C, Foxe JJ, Murray MM (2009). Early, low-level auditory-somatosensory multisensory interactions impact reaction time speed.. Front Integr Neurosci.

[73] Merabet LB, Hamilton R, Schlaug G, Swisher JD, Kiriakopoulos ET (2008). Rapid and reversible recruitment of early visual cortex for touch.. PLoS One.

[74] Barone P (2010). Is the primary visual cortex multisensory? Comment on “Crossmodal influences on visual perception” by Prof. Ladan Shams.. Phys Life Rev.

[75] Kaas JH, Hackett TA (2000). How the visual projection map instructs the auditory computational map.. J Comp Neurol.

[76] Rauschecker JP, Tian B (2000). Mechanisms and streams for processing of “what” and “where” in auditory cortex.. Proc Natl Acad Sci U S A.

[77] Rauschecker JP (1991). Mechanisms of visual plasticity: Hebb synapses, NMDA receptors, and beyond.. Physiol Rev.

[78] Smiley JF, Hackett TA, Ulbert I, Karmas G, Lakatos P (2007). Multisensory convergence in auditory cortex, I. Cortical connections of the caudal superior temporal plane in macaque monkeys.. J Comp Neurol.

[79] Hackett TA, Stepniewska I, Kaas JH (1999). Prefrontal connections of the parabelt auditory cortex in macaque monkeys.. Brain Res.

[80] Pandya DN, Hallett M, Kmukherjee SK (1969). Intra- and interhemispheric connections of the neocortical auditory system in the rhesus monkey.. Brain Res.

[81] Strelnikov K, Rouger J, Lagleyre S, Fraysse B, Deguine O (2009). Improvement in speech-reading ability by auditory training: Evidence from gender differences in normally hearing, deaf and cochlear implanted subjects.. Neuropsychologia.

[82] Rouger J, Lagleyre S, Fraysse B, Deneve S, Deguine O (2007). Evidence that cochlear-implanted deaf patients are better multisensory integrators.. Proc Natl Acad Sci U S A.

[83] Giraud AL, Price CJ, Graham JM, Truy E, Frackowiak RS (2001). Cross-modal plasticity underpins language recovery after cochlear implantation.. Neuron.

[84] Grantham DW, Ricketts TA, Ashmead DH, Labadie RF, Haynes DS (2008). Localization by postlingually deafened adults fitted with a single cochlear implant.. Laryngoscope.

[85] Frassinetti F, Pavani F, Ladavas E (2002). Acoustical vision of neglected stimuli: interaction among spatially converging audiovisual inputs in neglect patients.. J Cogn Neurosci.

[86] Leo F, Bolognini N, Passamonti C, Stein BE, Ladavas E (2008). Cross-modal localization in hemianopia: new insights on multisensory integration.. Brain.

[87] Carpenter J, Bithell J (2000). Bootstrap confidence intervals: when, which, what? A practical guide for medical statisticians.. Stat Med.

[88] Swets JA, Green DM, Getty DJ, Swets JB (1978). Signal detection and identification at successive stages of observation.. Percept Psychophys.

